# Associations of combined polygenic risk score and glycemic status with atrial fibrillation, coronary artery disease and ischemic stroke

**DOI:** 10.1186/s12933-023-02021-0

**Published:** 2024-01-03

**Authors:** Juntae Kim, Dongmin Kim, Han-Joon Bae, Byoung-Eun Park, Tae Soo Kang, Seong-Hoon Lim, Su Yeon Lee, Young Hak Chung, Ji Wung Ryu, Myung-Yong Lee, Pil-Sung Yang, Boyoung Joung

**Affiliations:** 1 Division of Cardiology, Department of Internal Medicine, College of Medicine, Dankook University, 119, Dandae-ro, Dongnam-gu, Cheonan-si, Chungnam, 31116 Republic of Korea; 2 Department of Cardiology, Daegu Catholic University Medical Center, 33 Duryugongwonro 17- gil, Nam-gu, Daegu, 42472 Republic of Korea; 3 Department of Cardiology, CHA Bundang Medical Center, CHA University, 59, Yatap-ro, Bundang-gu, Seongnam, 13496 Gyeonggi-do Republic of Korea; 4 Division of Cardiology, Department of Internal Medicine, Yonsei University College of Medicine, 50 Yonseiro, Seodaemun-gu, Seoul, 03722 Republic of Korea

**Keywords:** Polygenic risk score, Diabetes Mellitus, Hemoglobin A1c, Cardiovascular Disease, Atrial fibrillation, Coronary artery Disease, Ischemic Stroke

## Abstract

**Background:**

It is unknown whether high hemoglobin A1c (HbA1c) is associated with increases in the risk of cardiovascular disease among individuals with elevated genetic susceptibility. We aimed to investigate the association between HbA1c and atrial fibrillation (AF), coronary artery disease (CAD), and ischemic stroke according to the polygenic risk score (PRS).

**Methods:**

The UK Biobank cohort included 502,442 participants aged 40–70 years who were recruited from 22 assessment centers across the United Kingdom from 2006 to 2010. This study included 305,605 unrelated individuals with available PRS and assessed new-onset AF, CAD, and ischemic stroke. The participants were divided into tertiles based on the validated PRS for each outcome. Within each PRS tertiles, the risks of incident events associated with HbA1c levels were investigated and compared with HbA1c < 5.7% and low PRS. Data were analyzed from November 2022 to May 2023.

**Results:**

Of 305,605 individuals, 161,605 (52.9%) were female, and the mean (SD) age was 56.6 (8.1) years. During a median follow-up of 11.9 (interquartile range 11.1–12.6) years, the incidences of AF, CAD, and ischemic stroke were 4.6, 2.9 and 1.1 per 100 person-years, respectively. Compared to individuals with HbA1c < 5.7% and low PRS, individuals with HbA1c ≥ 6.5% and high PRS had a 2.67-times higher risk for AF (hazard ratio [HR], 2.67; 95% confidence interval (CI), 2.43–2.94), 5.71-times higher risk for CAD (HR, 5.71; 95% CI, 5.14–6.33) and 2.94-times higher risk for ischemic stroke (HR, 2.94; 95% CI, 2.47–3.50). In the restricted cubic spline models, while a U-shaped trend was observed between HbA1c and the risk of AF, dose-dependent increases were observed between HbA1c and the risk of CAD and ischemic stroke regardless PRS tertile.

**Conclusions:**

Our results suggest that the nature of the dose-dependent relationship between HbA1c levels and cardiovascular disease in individuals with different PRS is outcome-specific. This adds to the evidence that PRS may play a role together with glycemic status in the development of cardiovascular disease.

**Supplementary Information:**

The online version contains supplementary material available at 10.1186/s12933-023-02021-0.

## Introduction

Cardiovascular disease is a leading cause of mortality and a major public health issue worldwide. Although the burden of cardiovascular disease has decreased over the past decades, death caused by diabetes is still increasing [1]. Diabetes is an established risk factor for cardiovascular disease, and individuals with diabetes have an increased risk of cardiovascular mortality compared to those without diabetes [2]. Also, elevated fasting blood glucose and hemoglobin A1c (HbA1c) levels are associated with cardiovascular disease and mortality [3, 4]. Several studies have shown that glycemic status is associated with an increased risk of coronary artery disease (CAD) and ischemic stroke [5–7].

Atrial fibrillation (AF) increases the risk of mortality and morbidity resulting from stroke and heart failure, and also impairs the quality of life [8–10]. The incidence of AF is correlated with increasing age, obesity, hypertension, diabetes, heart failure, valvular heart disease, and CAD [11, 12]. In healthy individuals, high-normal blood pressure and impaired fasting glucose were important risk factors for AF [13]. Several studies have suggested that the duration of diabetes or insulin treatment were related to the development of AF [14, 15]. However, the evidence for an association between diabetes or glycemic status measured by HbA1c and risk of AF is still controversial [12, 16–20].

Polygenic risk scores (PRSs) provide personalized estimates of genetic information related to diseases. Several recent genome-wide association studies (GWASs) have demonstrated genetic variants associated with AF, CAD and ischemic stroke [21–23]. However, there are concerns about the methods to incorporate genetic variants to calculate the PRS and potential risks caused by inappropriate and biased information [24]. Recently, Thompson et al. [25] presented PRSs for several diseases, including AF, CAD, and ischemic stroke, in UK Biobank participants and released a PRS comparison tool to enable performance evaluation for different PRSs. It is currently unknown whether high HbA1c is associated with an increased risk of cardiovascular disease among individuals with elevated genetic susceptibility. This study aimed to evaluate the associations of high HbA1c and diabetes status with incident cardiovascular disease among individuals with different PRS and to investigate how PRSs interact with these associations in the UK Biobank.

## Method

### Study population

**Fig. 1:**
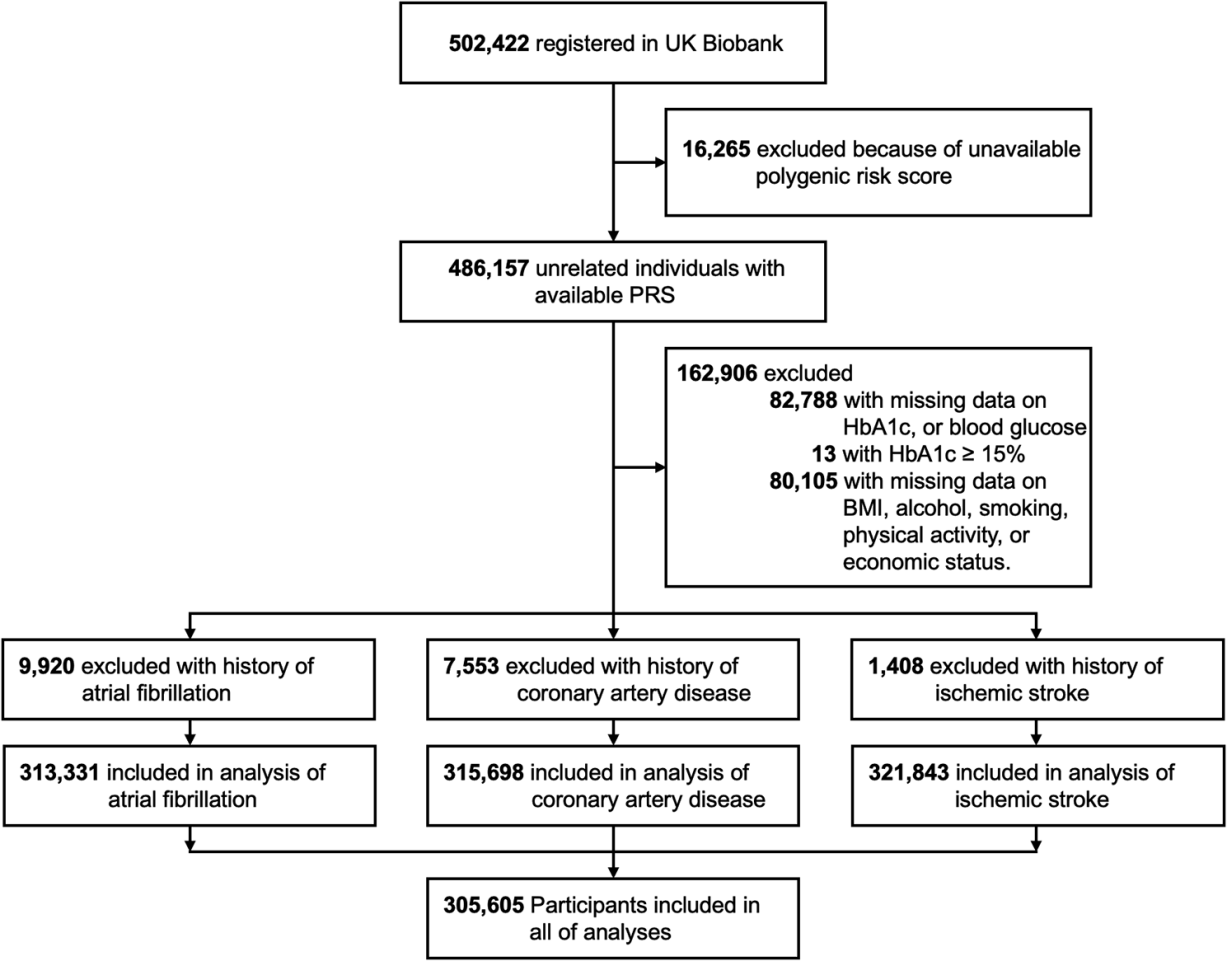
Flowchart for the selection of the analyzed study sample from the UK Biobank study. AF, atrial fibrillation; BMI, body mass index; HbA1c, hemoglobin A1c; PRS, polygenic risk score

The UK Biobank is a large-scale database consisting of more than 500,000 participants aged 40–69 years from 22 assessment centers throughout the United Kingdom between 2006 and 2010. The participants had undergone an extensive range of physical measures, provided information on their environment, lifestyle, medical history, and genetic data at recruitment, and agreed to have their health information followed up through linkages to electronic health-related records. The details of the study design and data collection have been described previously [26]. This study included the UK Biobank participants with measurement of HbA1c at enrollment, HbA1c < 15%, and available genetic data [25]. Participants with prevalent disease outcome were excluded in each analysis (Fig. 1). UK Biobank received ethical approval from the Northwest Multicenter Research Ethics Committee. The UK Biobank data are available to researchers after acceptance of the research proposal to the UK Biobank. Written informed consent was obtained from all the participants during recruitment. This study has been conducted using the UK Biobank Resource (Application No 77,793). This study was approved by the Institutional Review Board of Yonsei University Health System (4-2023-0323).

From the 486,157 unrelated individuals with available PRS data, 162,906 participants were excluded based on the following criteria: (1) 82,788 with missing data on HbA1c, or blood glucose, (2) 13 with HbA1c ≥ 15%, (3) 80,105 with missing data on body mass index, alcohol, smoking, physical activity, or economic status. Participants with prevalent diseases were excluded from each analysis (Fig. 1), leaving 313,331 participants for the analysis of AF; 315,698 for CAD; and 321,843 for ischemic stroke. After these exclusions, 305,605 participants were included in all of analyses.

### Polygenic risk score

The PRSs available from the UK Biobank were generated by other researchers and reviewed by UK Biobank. PRS were calculated as the sum of the effect sizes of individual genetic variants multiplied by the allele dosage. The effect sizes of the single nucleotide polymorphism-disease associations were based on external GWAS data, which were estimated via a fixed-effect inverse variance meta-analysis. Details for generating the PRS have been previously described [25]. The PRS were then divided into tertiles to categorize individuals into low, intermediate, and high. In addition, we conducted a sensitivity analysis using the PRS categorized as low (quintile 1), intermediate (quintiles 2–4), or high (quintile 5).

### Glycemic status and covariates

Glycemic status was evaluated using HbA1c (mmol/mol), which was measured through high-performance liquid chromatography analysis on a Bio-Rad VARIANT II Turbo using non-fasting blood samples [27]. The unit in mmol/mol was converted to percentage (%) based on the equation: (0.09148 × HbA1c in mmol/mol) + 2.152 [28]. HbA1c was classified as < 5.7%, 5.7–6.4%, and ≥ 6.5%. The diabetes status was classified as diabetes, prediabetes, or normoglycemia. Diabetes identified through self-reported medical conditions or diagnosis using the ICD-10 code (E10-14), and/or the use of insulin treatment. Undiagnosed patients were identified, according to the ADA criteria, based on HbA1c ≥ 6.5%.^29^ Prediabetes was defined as no diagnosis of diabetes, and HbA1c ≥ 5.7 and < 6.5%. Normoglycemia was defined as no diagnosis of diabetes or an HbA1c < 5.7%.

Ethnicity was self-reported and categorized as Asian, Black, White, or Mixed. Participants reported their history of smoking status (non-, ex-, current smokers), frequency of alcohol intake (non-, 1–3 times/month, 1–2 times/week, and ≥ 3 times/week). Physical activity was assessed using the International Physical Activity Questionnaire (IPAQ) and was categorized as low, moderate, or high. The Townsend Deprivation Index was divided into five categories based on UK census data.

### Outcomes

Disease outcomes were defined as self-reported medical conditions or the first event occurring during at least two different days of hospital visits (primary care data) or the first admission (hospital inpatient data) with the ICD-10 code. Detailed definitions of the outcomes and comorbidities are presented in eTables 1 and 2. Hospital registry-based follow-up ended on March 31, 2021, in England and Scotland, and February 28, 2018, in Wales. The cohorts were followed up until the occurrence of each outcome, death, emigration, or the end of the study, whichever occurred first.

### Statistical analysis

Multivariate Cox proportional hazards regression analysis was performed to test the association of the PRS and HbA1c with incident AF, CAD and ischemic stroke. Individuals with low PRS and HbA1c levels of < 5.7% or normoglycemia were used as the reference group in each analysis. Cox regression analyses were adjusted for age; sex; ethnicity; body mass index; smoking; alcohol consumption; hypertension; dyslipidemia; heart failure; peripheral artery disease; chronic kidney disease; end-stage renal disease; physical activity; and economic status. The proportional hazards assumption was verified using Schoenfeld residuals. The Wald test was used to analyze the differences between the hazard ratios (HR) of each exposure within the PRS tertiles (P_difference_). Restricted cubic splines were used to estimate the potential nonlinearity of the associations between HbA1c levels and outcomes according to PRS tertiles. The number of knots for the restricted cubic spline models was selected using the Bayesian information criterion (BIC). The reference value for the spline curve was 5.5% of HbA1c. We used the Bonferroni correction to adjust for multiple testing and considered 2-sided P values < 0.017 (P value < 0.05, divided by the number of tests, i.e., 0.05/3) statistically significant. Statistical analyses were conducted using R software (version 4.3.3; R Foundation, www.R-project.org).

## Results

### Population characteristics

Of 306,605 individuals, 161,605 (52.9%) were female, and the mean (SD) age was 56.6 (8.1) years. 251,947 (82.4%) had HbA1c < 5.7%, 43,307 (14.2%) had HbA1c 5.7–6.4%, and 10,351 (3.4%) had HbA1c ≥ 6.5%. A total of 250,468 (81.9%) patients had normoglycemia, 41,522 (13.6%) had prediabetes, and 13,615 (4.5%) had diabetes. Depending on the outcome, the size of the study population varied with the prevalence of disease exclusion. Baseline characteristics according to HbA1c category are presented as PRS tertiles of AF (eTable 3), CAD (eTable 4), and ischemic stroke (eTable 5). In general, individuals with higher HbA1c levels were more likely to be male, older, have a higher body mass index, and more comorbidities, including hypertension, dyslipidemia, heart failure, peripheral artery disease, chronic kidney disease, and end-stage renal disease, than individuals with lower HbA1c levels (Table 1).

**Table 1:** Baseline characteristics

Characteristics	HbA1c < 5.7% (n = 251,947)	HbA1c 5.7–6.4% (n = 43,307)	HbA1c ≥ 6.5% (n = 10,351)	P value
Age, mean (SD), y	55.9 ± 8.2	59.8 ± 7.1	59.3 ± 7.3	< 0.001
Female	135,011 (53.6%)	22,735 (52.5%)	3859 (37.3%)	< 0.001
Ethnicity				< 0.001
White	242,133 (96.1%)	39,208 (90.5%)	8954 (86.5%)	
Black	2264 (0.9%)	1282 (3.0%)	345 (3.3%)	
Asian	3777 (1.5%)	1765 (4.1%)	747 (7.2%)	
Mixed/other	3773 (1.5%)	1052 (2.4%)	305 (2.9%)	
BMI, mean (SD)	26.8 ± 4.3	28.9 ± 5.2	31.5 ± 5.8	< 0.001
Alcohol				< 0.001
Non	15,887 (6.3%)	4519 (10.4%)	1573 (15.2%)	
1–2 times/week	117,029 (46.4%)	22,260 (51.4%)	5603 (54.1%)	
≥ 3 times/week	119,031 (47.2%)	16,528 (38.2%)	3175 (30.7%)	
Smoking				< 0.001
Non-	142,872 (56.7%)	21,326 (49.2%)	4936 (47.7%)	
Ex-	85,423 (33.9%)	15,602 (36.0%)	4168 (40.3%)	
Current-	23,652 (9.4%)	6379 (14.7%)	1247 (12.0%)	
Hypertension	55,261 (21.9%)	16,312 (37.7%)	6217 (60.1%)	< 0.001
Dyslipidemia	24,540 (9.7%)	9865 (22.8%)	4121 (39.8%)	< 0.001
Heart failure	4341 (1.7%)	1014 (2.3%)	310 (3.0%)	< 0.001
Peripheral artery disease	742 (0.3%)	238 (0.5%)	74 (0.7%)	< 0.001
CKD or ESRD	1766 (0.7%)	656 (1.5%)	303 (2.9%)	< 0.001
Physical activity				< 0.001
Low	45,067 (17.9%)	8958 (20.7%)	2982 (28.8%)	
Intermediate	102,849 (40.8%)	17,754 (41.0%)	4128 (39.9%)	
High	104,031 (41.3%)	16,595 (38.3%)	3241 (31.3%)	
Economic status				< 0.001
Q1 (Lowest)	30,264 (12.0%)	6702 (15.5%)	2142 (20.7%)	
Q2	32,262 (12.8%)	6132 (14.2%)	1661 (16.0%)	
Q3	37,605 (14.9%)	6534 (15.1%)	1536 (14.8%)	
Q4	52,967 (21.0%)	8717 (20.1%)	1891 (18.3%)	
Q5 (Highest)	98,849 (39.2%)	15,222 (35.1%)	3121 (30.2%)	

Values are mean ± SD, or No. (%)

BMI = body mass index, CD = chronic kidney disease, ESRD = end stage renal disease, SD = standard deviation

### Association of PRS with incident cardiovascular disease

During a median (interquartile range) follow-up of 11.9 (11.1–12.6) years, 17,397 participants developed AF, 12,895 developed CAD and 3,489 developed ischemic stroke. eFigure 1 shows that a higher PRS was associated with a higher incidence of AF, CAD, and ischemic stroke during follow-up. In multivariable adjusted models, high PRS was associated with a higher risk of incident AF (HR, 2.46; 95% CI, 2.37–2.56; P < .001), CAD (HR, 3.26; 95% CI, 3.09–3.44; P < .001), ischemic stroke (HR, 1.57; 95% CI, 1.45–1.71; P < .001) compared with low PRS (eTable 6). The influence of the PRS tertiles of each outcome on incident AF, CAD, and ischemic stroke was comparable with other clinical risk factors, including diabetes, hypertension, heart failure, peripheral artery disease, and chronic kidney disease (eFigure 2).

### Association of HbA1c and PRS with incident cardiovascular disease

**Fig. 2:**
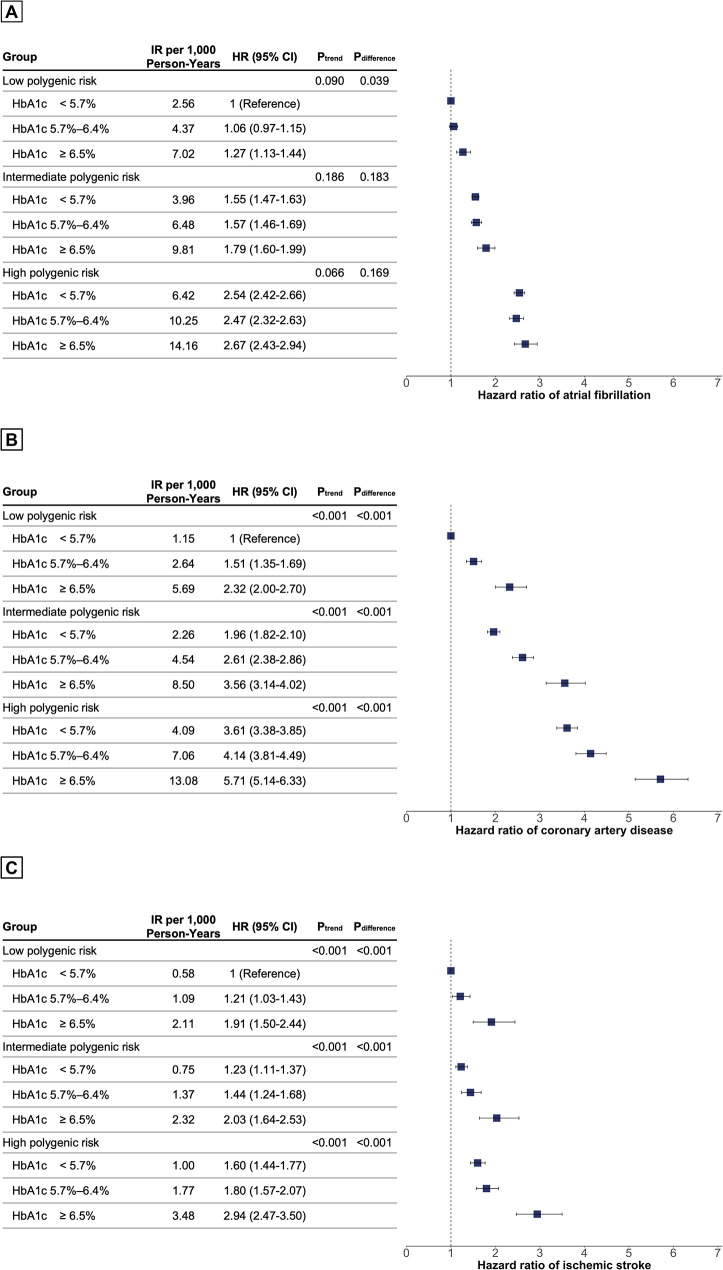
Associations of hemoglobin A1c with atrial fibrillation, coronary artery disease, and ischemic stroke by polygenic risk score tertiles. Adjusted for age, sex, ethnicity, body mass index, smoking, alcohol, hypertension, dyslipidemia, heart failure, peripheral artery disease, chronic kidney disease, end stage renal disease, physical activity, and economic status. Hazard ratios (HRs) are provided with 95% CIs. The vertical line indicates the reference value of 1.

Compared with HbA1c < 5.7% and low PRS, individuals with HbA1c ≥ 6.5% and high PRS were associated with high risk of AF (HR, 2.67; 95% CI 2.43–2.94; P < .001), CAD (HR, 5.71; 95% CI 5.14–6.33; P < .001) and ischemic stroke (HR, 2.94; 95% CI 2.47–3.50; P < .001) (Fig. 2). HbA1c > 6.5% was gradually associated with an increased risk of CAD (Fig. 2B) and ischemic stroke (Fig. 2C) across all PRS tertiles (P_trend_ < 0.001 and P_difference_ < 0.001, respectively). However, the association between HbA1c > 6.5% and AF was significant only in individuals with a low PRS. (Fig. 2A).

**Fig. 3:**
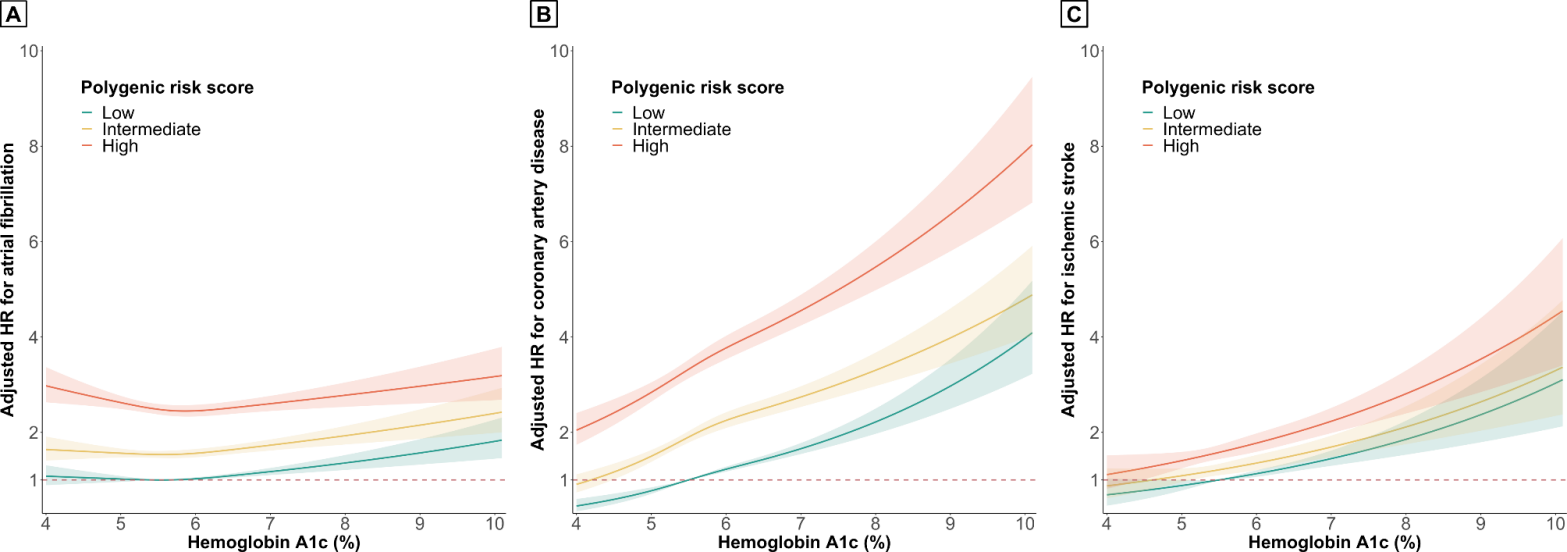
Nonlinear dose–response analysis of hemoglobin A1c and the risk of atrial fibrillation, coronary artery disease and ischemic stroke, by polygenic risk score tertiles. The model is centered at 5.5% HbA1c with knots at 5.0%, 5.7%, and 6.5% HbA1c.

In the restricted cubic spline models, the risk of AF decreased to a nadir at 5.5% of HbA1c and then increased (P_nonlinearity_ < 0.001). The risks of CAD and ischemic stroke increased steadily with increasing HbA1c levels. However, compared with HbA1c < 5.7%, the effect of HbA1c on CAD risk was modest at HbA1c ≥ 5.7% (P_nonlinearity_ < 0.001). In individuals with low PRS, the HR of AF, CAD, and ischemic stroke at 9% HbA1c were 1.33 (95% CI 1.22–1.45), 2.19 (95% CI 2.01–2.38) and 2.27 (95% CI 1.96–2.64) respectively, compared with 5.5% HbA1c (Fig. 3).

### Associations of diabetes status and PRS with incident cardiovascular disease

**Fig. 4:**
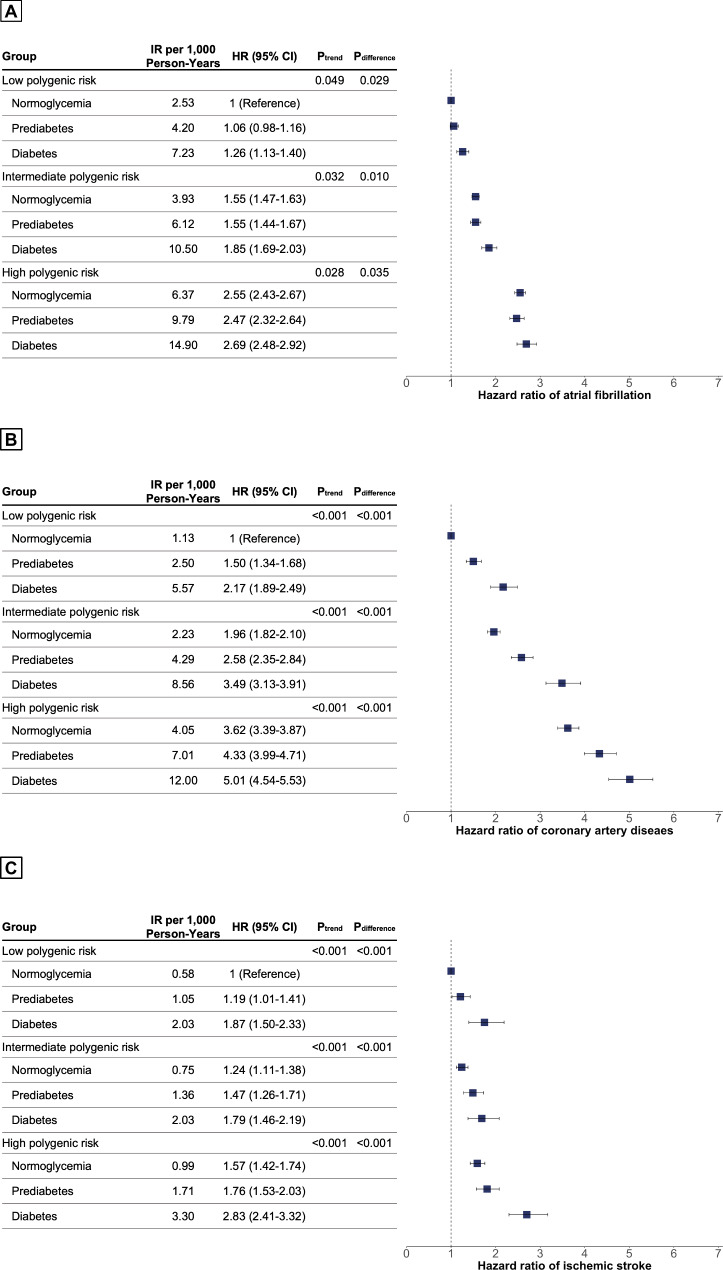
Associations of diabetes status with atrial fibrillation, coronary artery disease and ischemic stroke by polygenic risk score tertiles. Adjusted for age, sex, ethnicity, body mass index, smoking, alcohol, hypertension, dyslipidemia, heart failure, peripheral artery disease, chronic kidney disease, end stage renal disease, physical activity, and economic status. Hazard ratios (HRs) are provided with 95% CIs. The vertical line indicates the reference value of 1.

Compared with normoglycemia and low PRS, individuals with diabetes and high PRS were associated with high risk of AF (HR, 2.69; 95% CI 2.48–2.92; P < .001), CAD (HR, 5.01; 95% CI 4.54–5.53; P < .001) and ischemic stroke (HR, 2.83; 95% CI 2.41–3.32; P < .001) (Fig. 4). Diabetes was associated with a higher risk of CAD (Fig. 4B) and ischemic stroke (Fig. 4C) in each PRS tertile (P_trend_ < 0.001 and P_difference_ < 0.001, respectively). However, the association between diabetes status and a higher risk of AF was significant only in the intermediate PRS tertile (P_difference_ = 0.010).

### Sensitivity analysis

In the sensitivity analysis, the results with PRS categorized as low (quintile 1), intermediate (quintile 2–4), or high (quintile 5) remained essentially unchanged (eFigure 3). The analyses on the European population did not indicate differential associations between HbA1c and all outcomes (eFigure 4–5). There were no significant interactions by sex in the association of HbA1c levels with AF or ischemic stroke, regardless of the PRS tertiles. However, the association between HbA1c levels and CAD showed significant interactions with sex, especially in the high PRS tertile (eFigure 6).

## Discussion

Using a large-scale, population-based cohort of more than 300,000 individuals, we analyzed the association between HbA1c and cardiovascular disease in individuals with different PRSs for each outcome. Our major findings include the following: (i) PRS was associated with the risk of new-onset AF, CAD, and ischemic stroke. (ii) HbA1c > 6.5% was associated with a higher risk of CAD and ischemic stroke across all polygenic risk tertiles, but not with AF. In restricted cubic spline models, the presence of a gradual increase in the risk of CAD and ischemic stroke across HbA1c levels was evident, but a U-shaped trend was observed for AF in all polygenic risk tertiles. (iii) Diabetes status was also strongly associated with a higher risk of CAD and ischemic stroke across all polygenic risk tertiles, but not with AF. Our results suggest that the nature of the dose-dependent relationship between HbA1c levels and cardiovascular disease in individuals with different PRS is outcome-specific.

### Glycemic status and cardiovascular disease

Previous studies have shown that chronic hyperglycemia, reflected by HbA1c levels, is associated with a higher risk of cardiovascular disease [20, 30, 31]. However, there are different associations between glycemic status and AF, CAD, and ischemic stroke. Eoin et al. [32] reported that higher HbA1c levels at the time of AF ablation increased post-ablation recurrence rates. The duration of diabetes and insulin use are associated with a higher risk of AF and an elevated thromboembolic risk in patients with AF [14, 15, 33, 34]. Zhang et al. [35] suggested diabetes is an independent risk factor for stroke recurrence among patients with ischemic stroke. Tight glucose control using insulin after an acute ischemic stroke is not associated with an improved prognosis [36]. Long-term insulin treatment did not improve survival in type 2 diabetic patients following myocardial infarction [37]. Shahim et al. [38] found that 2-hour plasma glucose value during a 75-g oral glucose tolerance test, but not HbA1c, added important prognostic information regarding future cardiovascular events. Also, mendelian randomization analyses support a causal link between diabetes and a higher risk of CAD and ischemic stroke but not AF [19, 39, 40].

### Values of glycemic status in patient with different genetic traits

Although the performance of the PRS differs across cardiovascular diseases, a higher PRS of AF, CAD, or ischemic stroke has been found to be associated with an increased risk of new-onset AF, CAD, or ischemic stroke. However, it is unknown whether high HbA1c is associated with an increase in the risk of cardiovascular disease among individuals with different genetic susceptibilities for cardiovascular disease. In this study, HbA1c > 6.5% was significantly associated with an increased risk of CAD and ischemic stroke across all PRS tertiles. Our findings imply the strong potential benefits of lowering HbA1c levels to prevent cardiovascular diseases, regardless of PRS. The exact mechanisms underlying the association between HbA1c levels, PRS, and cardiovascular diseases are unknown. Several mechanisms may underlie the interaction between HbA1c and PRS. In this study, individuals with a higher PRS for cardiovascular diseases had more comorbidities. Recent studies have demonstrated that CAD, ischemic stroke and type 2 diabetes share a genetic background with each other and with their commonly associated risk factors [41]. Further studies are necessary to investigate the potential of optimal glycemic control based on PRS to prevent cardiovascular disease, as a randomized controlled trials demonstrated that statin therapy led to a greater relative risk reduction among individuals with high PRS of coronary heart disease [42].

Diabetes is a risk factor for the development of AF and may be related to its underlying pathogenesis, including oxidative stress, increased non-enzymatic glycosylation, structural and electrical remodeling, and autonomic dysfunction [43, 44]. Recent meta-analyses have reported an association between higher HbA1c levels and an increased risk of AF [18, 20]. However, in our study, the association between HbA1c levels and AF was insignificant in each PRS tertile. As the PRS increases, the effects of HbA1c on AF tend to decrease. The risk of AF associated with elevated HbA1c may appear to be mediated by diabetes-related comorbidities, such as hypertension and obesity [19, 45]. Likewise, several studies have suggested that low HbA1c is a marker of underlying diseases, such as hemoglobinopathy, chronic liver disease, cardiovascular mortality, and all-cause mortality [30, 46–48]. This hypothesis may be consistent with our results regarding the association between low HbA1c and increased risk of AF.

### Strengths and limitations

The major strengths of this study were the use of a large-scale database with prospective ascertainment of outcomes and the validated PRS to facilitate subsequent research with reproducible findings. This study had several limitations. First, the UK Biobank participants were not representative of the general UK population. Due to healthy volunteer selection bias, participants differed in sociodemographic, lifestyle, and health-related characteristics from the general population [26]. However, a generalizable association between exposure and health conditions can be estimated using a sufficiently large number of participants with different levels of exposure. Second, the PRS was generated primarily based on GWAS data from individuals of European ancestry, which may be inappropriate for application to other racial and ethnic groups. Further GWAS data on individuals of non-European ancestry are needed to reduce the performance differences across ancestries. Third, there are differences in phenotype definitions between the study outcomes and the PRS. Nevertheless, in this study, the PRS has sufficient predictive performance. Fourth, the outcomes and comorbidities based on self-reported data might be susceptible to misclassification errors. These errors tend to be biased toward the null hypothesis and underestimate the association between exposure and outcomes. Finally, the trajectory of HbA1c levels during the follow-up period was not considered. Repeated exposure measurements over time may adequately represent the actual health-related conditions. Although individuals with HbA1c ≥ 15% were excluded to minimize the possibility of hemoglobinopathies, other conditions associated with falsely elevated or falsely lowered A1c were not excluded [29, 49].

## Conclusion

Elevated HbA1c was associated with an increased risk of CAD and ischemic stroke across all polygenic risk tertiles. However, high HbA1c did not increased the risk of AF in patients with high PRS of AF. Our results suggest that the nature of the dose-dependent relationship between HbA1c levels and cardiovascular disease in individuals with different PRS is outcome-specific. This adds to the evidence that PRS may play a role together with glycemic status in the development of cardiovascular disease.

### Electronic supplementary material

Below is the link to the electronic supplementary material.

Supplementary Material 1

## Data Availability

All researchers in academic, commercial and charitable settings can apply to use the UK Biobank resource for health-related research in the public interest (www.ukbiobank.ac.uk/registerapply/). Details for generating the PRS is available at https://www.medrxiv.org/content/10.1101/2022.06.16.22276246v2.

## Abbreviations

AF: Atrial fibrillation
CAD: Coronary artery disease
HR: Hazard ratios
HbA1c: Hemoglobin A1c
PRS: Polygenic risk score
