# Supplementary material for: Associations of combined polygenic risk score and glycemic status with atrial fibrillation, coronary artery disease and ischemic stroke
Source: Cardiovasc Diabetol. 2024 Jan 3;23:5. doi: 10.1186/s12933-023-02021-0 (PMC10765629; doi:10.1186/s12933-023-02021-0)

**ONLINE SUPPLEMENTARY CONTENTS**

**Associations of combined polygenic risk score and glycemic status with atrial fibrillation, coronary artery disease and ischemic stroke in the UK Biobank**

**eTable 1.** Definitions used for defining the outcomes.

**eTable 2.** Definitions used for defining the comorbidities.

**eTable 3.** Baseline characteristics by glycemic status and polygenic risk group of atrial fibrillation.

**eTable 4.** Baseline characteristics by glycemic status and polygenic risk group of coronary artery disease.

**eTable 5.** Baseline characteristics by glycemic status and polygenic risk group of ischemic stroke.

**eTable 6.** Cumulative incidence and incidence rates for outcomes according to polygenic risk score group.

**eFigure 1.** Incidence rate of atrial fibrillation, coronary artery disease and ischemic stroke according to the percentile of the polygenic score.

**eFigure 2.** Hazard ratios for atrial fibrillation, coronary artery disease and ischemic stroke by polygenic risk score and clinical risk factors.

**eFigure 3.** Associations of hemoglobin A1c with atrial fibrillation, coronary artery disease and ischemic stroke by polygenic risk score, categorized as low (quintile 1), intermediate (quintile 2-4), or high (quintile 5): sensitivity analysis.

**eFigure 4.** Associations of hemoglobin A1c with atrial fibrillation, coronary artery disease and ischemic stroke by polygenic risk score in the European population.

**eFigure 5.** Nonlinear dose–response analysis of hemoglobin A1c and the risk of atrial fibrillation, coronary artery disease and ischemic stroke, by polygenetic risk score tertiles in the European and Asian population.

**eFigure 6.** Risk of incident atrial fibrillation, coronary artery disease and ischemic stroke associated with polygenic risk score and lifestyle stratified by sex.

**eTable 1.** Definitions used for defining the outcomes.

| **Comorbidities** | **Definitions** | **Used codes or conditions** |
| --- | --- | --- |
| New onset atrial fibrillation (AF) | Defined from UK Biobank self-report or diagnosis* | Self-reported non-cancer illness code: 1471, 1483  ICD-10: I48 |
| Coronary artery disease | Defined from UK Biobank self-report or diagnosis* | Self-reported non-cancer illness code: 1075  ICD-10: I21, I22, I25.2 |
| Ischemic stroke | Defined from UK Biobank self-report or diagnosis* | Self-reported non-cancer illness code: 1583  ICD-10: I63, I64 |

***To ensure accuracy, comorbidities were established based on more than one hospital-inpatient or two outpatient (= primary care in United Kingdom) records of ICD-10 codes in the database.**

**eTable 2.** Definitions used for defining the comorbidities.

| **Comorbidities** | **Definitions** | **Used codes or conditions** |
| --- | --- | --- |
| Hypertension | Defined from UK Biobank self-report or diagnosis* | Self-reported non-cancer illness code: 1065, 1072  ICD-10: I10, I11, I12, I13, I15 |
| Dyslipidemia | Defined from UK Biobank self-report or diagnosis* | Self-reported non-cancer illness code: 1473  ICD-10: E78 |
| Heart failure | Defined from UK Biobank self-report or diagnosis* | Self-reported non-cancer illness code: 1076  ICD-10: I11.0, I50, I97.1 |
| Peripheral arterial disease | Defined from UK Biobank self-report or diagnosis* | Self-reported non-cancer illness code: 1067, 1087  ICD-10: I70, I71 |
| Chronic kidney disease | Defined from eGFR (if laboratory value was not available, self-report or diagnosis code was used) | eGFR <60 mL/min per 1.73 m^2^  Self-reported non-cancer illness code:1192, 1194  ICD-10: N18, N19 |
| ESRD | Defined from UK Biobank self-report or procedure codes related to renal replacement therapy (hemodialysis, peritoneal dialysis, or kidney transplant) | Self-reported non-cancer illness code: 1193, 1195, 1580, 1581, 1582  Procedure codes: L74, M01, M02.3, M08.4, M17, X40, X41, X42 |

***To ensure accuracy, comorbidities were established based on more than one hospital-inpatient or two outpatient (= primary care in United Kingdom) records of ICD-10 codes in the database.**

**eTable 3. Baseline characteristics by glycemic status and polygenic risk group of atrial fibrillation.**

|  | **Low PRS** | | | **Intermediate PRS** | | | **High PRS** | | |
| --- | --- | --- | --- | --- | --- | --- | --- | --- | --- |
|  | **HbA1c < 5.6%** | **HbA1c 5.7-6.4%** | **HbA1c ≥ 6.5%** | **HbA1c < 5.6%** | **HbA1c 5.7-6.4%** | **HbA1c ≥ 6.5%** | **HbA1c < 5.6%** | **HbA1c 5.7-6.4%** | **HbA1c ≥ 6.5%** |
| Total, No. | 85623 | 14796 | 5090 | 85181 | 14503 | 5018 | 83874 | 14230 | 5016 |
| Age, mean (SD), y | 56.0 ± 8.2 | 59.9 ± 7.1 | 59.7 ± 7.2 | 56.0 ± 8.2 | 60.0 ± 7.0 | 59.6 ± 7.3 | 55.9 ± 8.2 | 60.0 ± 7.1 | 59.4 ± 7.3 |
| Female | 47.4% | 49.5% | 64.7% | 47.0% | 48.9% | 64.9% | 46.5% | 48.9% | 64.1% |
| Ethnicity |  |  |  |  |  |  |  |  |  |
| White | 95.4% | 88.6% | 84.6% | 96.4% | 91.6% | 87.6% | 96.6% | 91.9% | 88.2% |
| Black | 1.1% | 3.5% | 3.9% | 0.8% | 2.4% | 3.0% | 0.8% | 2.6% | 2.5% |
| Asian | 1.8% | 5.1% | 8.2% | 1.4% | 3.7% | 6.8% | 1.3% | 3.4% | 6.6% |
| Mixed/other | 1.7% | 2.7% | 3.4% | 1.4% | 2.3% | 2.6% | 1.3% | 2.1% | 2.7% |
| BMI, mean (SD) | 26.7 ± 4.3 | 28.8 ± 5.2 | 31.4 ± 5.7 | 26.8 ± 4.3 | 28.9 ± 5.2 | 31.5 ± 5.7 | 26.9 ± 4.4 | 29.0 ± 5.2 | 31.6 ± 5.9 |
| Alcohol |  |  |  |  |  |  |  |  |  |
| Non | 6.4% | 11.3% | 15.0% | 6.3% | 10.5% | 15.9% | 6.3% | 9.8% | 15.1% |
| 1-2 times/week | 46.2% | 50.6% | 54.4% | 46.6% | 51.3% | 53.7% | 46.4% | 51.8% | 54.6% |
| ≥3 times/week | 47.4% | 38.1% | 30.6% | 47.1% | 38.2% | 30.4% | 47.2% | 38.4% | 30.3% |
| Smoking |  |  |  |  |  |  |  |  |  |
| Non- | 56.7% | 48.9% | 47.1% | 56.5% | 47.9% | 44.9% | 55.9% | 47.9% | 45.4% |
| Ex- | 34.2% | 36.7% | 40.7% | 34.1% | 37.4% | 43.6% | 34.3% | 36.5% | 41.4% |
| Current- | 9.1% | 14.5% | 12.3% | 9.4% | 14.7% | 11.5% | 9.8% | 15.6% | 13.2% |
| Hypertension | 20.9% | 37.2% | 61.2% | 22.8% | 39.1% | 61.1% | 24.1% | 41.2% | 63.8% |
| Dyslipidemia | 10.3% | 24.6% | 42.8% | 10.6% | 24.9% | 42.2% | 10.7% | 25.0% | 42.2% |
| Heart failure | 1.9% | 2.8% | 4.3% | 1.9% | 2.6% | 3.8% | 1.8% | 2.9% | 3.4% |
| Peripheral artery disease | 0.3% | 0.5% | 0.8% | 0.3% | 0.7% | 0.9% | 0.3% | 0.7% | 1.1% |
| CKD or ESRD | 0.7% | 1.7% | 3.2% | 0.7% | 1.6% | 3.4% | 0.8% | 1.8% | 3.1% |
| IPAQ |  |  |  |  |  |  |  |  |  |
| Low | 17.8% | 20.9% | 28.6% | 18.0% | 20.6% | 29.4% | 18.0% | 21.1% | 29.3% |
| Intermediate | 41.1% | 41.0% | 40.3% | 40.7% | 41.2% | 39.5% | 40.6% | 40.7% | 39.6% |
| High | 41.2% | 38.1% | 31.1% | 41.2% | 38.2% | 31.1% | 41.4% | 38.2% | 31.2% |
| Economic status |  |  |  |  |  |  |  |  |  |
| Q1 (Lowest) | 12.3% | 15.8% | 21.2% | 12.0% | 15.4% | 20.7% | 12.1% | 16.2% | 21.6% |
| Q2 | 12.9% | 14.0% | 15.9% | 12.7% | 14.5% | 16.9% | 12.8% | 14.2% | 15.7% |
| Q3 | 15.0% | 15.7% | 13.8% | 14.9% | 14.6% | 15.2% | 14.8% | 14.9% | 15.3% |
| Q4 | 21.0% | 20.0% | 18.5% | 21.0% | 19.9% | 17.9% | 21.1% | 20.0% | 18.2% |
| Q5 (Highest) | 38.9% | 34.5% | 30.5% | 39.3% | 35.6% | 29.3% | 39.2% | 34.8% | 29.2% |

Values are mean ± SD, or %

IPAQ = International Physical Activity Questionnaire

**eTable 4. Baseline characteristics by glycemic status and polygenic risk group of coronary artery disease.**

|  | **Low PRS** | | | **Intermediate PRS** | | | **High PRS** | | |
| --- | --- | --- | --- | --- | --- | --- | --- | --- | --- |
|  | **HbA1c < 5.6%** | **HbA1c 5.7-6.4%** | **HbA1c ≥ 6.5%** | **HbA1c < 5.6%** | **HbA1c 5.7-6.4%** | **HbA1c ≥ 6.5%** | **HbA1c < 5.6%** | **HbA1c 5.7-6.4%** | **HbA1c ≥ 6.5%** |
| Total, No. | 88566 | 14032 | 4323 | 86223 | 14556 | 4863 | 82889 | 14837 | 5409 |
| Age, mean (SD), y | 56.2 ± 8.2 | 56.0 ± 8.2 | 55.8 ± 8.2 | 60.2 ± 7.0 | 60.0 ± 7.1 | 59.7 ± 7.2 | 59.9 ± 7.1 | 59.5 ± 7.4 | 59.2 ± 7.3 |
| Female | 47.3% | 46.9% | 45.8% | 49.0% | 48.3% | 47.0% | 64.9% | 62.8% | 61.6% |
| Ethnicity |  |  |  |  |  |  |  |  |  |
| White | 96.3% | 96.4% | 95.7% | 91.1% | 91.6% | 89.7% | 87.1% | 88.3% | 85.7% |
| Black | 1.0% | 0.8% | 0.9% | 3.2% | 2.5% | 3.0% | 3.6% | 3.2% | 2.9% |
| Asian | 1.3% | 1.3% | 1.9% | 3.5% | 3.7% | 4.7% | 6.1% | 6.4% | 8.2% |
| Mixed/other | 1.5% | 1.5% | 1.5% | 2.3% | 2.3% | 2.6% | 3.2% | 2.1% | 3.2% |
| BMI, mean (SD) | 26.7 ± 4.3 | 26.8 ± 4.3 | 26.9 ± 4.4 | 28.8 ± 5.2 | 28.9 ± 5.3 | 29.1 ± 5.3 | 31.5 ± 5.8 | 31.5 ± 5.9 | 31.6 ± 5.9 |
| Alcohol |  |  |  |  |  |  |  |  |  |
| Non | 6.3% | 6.2% | 6.5% | 10.1% | 10.5% | 10.9% | 14.7% | 15.1% | 16.1% |
| 1-2 times/week | 46.1% | 46.3% | 46.8% | 51.2% | 50.9% | 51.8% | 53.9% | 54.1% | 53.9% |
| ≥3 times/week | 47.6% | 47.5% | 46.7% | 38.7% | 38.6% | 37.3% | 31.4% | 30.8% | 30.0% |
| Smoking |  |  |  |  |  |  |  |  |  |
| Non- | 57.1% | 56.5% | 56.1% | 48.5% | 48.8% | 49.3% | 46.1% | 47.3% | 48.0% |
| Ex- | 34.0% | 34.0% | 34.3% | 36.8% | 36.5% | 36.3% | 41.9% | 39.8% | 40.8% |
| Current- | 8.9% | 9.4% | 9.7% | 14.7% | 14.7% | 14.4% | 12.0% | 12.9% | 11.2% |
| Hypertension | 19.5% | 22.4% | 25.4% | 34.6% | 38.5% | 42.6% | 58.4% | 60.7% | 63.5% |
| Dyslipidemia | 7.9% | 9.8% | 12.6% | 19.9% | 23.0% | 27.4% | 37.5% | 39.7% | 44.2% |
| Heart failure | 1.8% | 1.8% | 2.0% | 2.6% | 2.6% | 2.9% | 3.6% | 3.8% | 3.4% |
| Peripheral artery disease | 0.3% | 0.3% | 0.4% | 0.5% | 0.6% | 0.7% | 0.6% | 1.0% | 0.9% |
| CKD or ESRD | 0.7% | 0.8% | 0.9% | 1.6% | 1.9% | 1.8% | 3.8% | 3.0% | 3.1% |
| IPAQ |  |  |  |  |  |  |  |  |  |
| Low | 17.9% | 17.8% | 18.1% | 20.4% | 20.9% | 20.9% | 29.6% | 28.1% | 29.7% |
| Intermediate | 41.0% | 41.1% | 40.3% | 41.7% | 40.7% | 40.8% | 39.8% | 40.3% | 39.4% |
| High | 41.0% | 41.2% | 41.5% | 37.9% | 38.4% | 38.3% | 30.6% | 31.6% | 31.0% |
| Economic status |  |  |  |  |  |  |  |  |  |
| Q1 (Lowest) | 12.0% | 11.8% | 12.4% | 15.0% | 15.5% | 16.0% | 19.5% | 20.5% | 21.8% |
| Q2 | 12.8% | 12.7% | 12.9% | 13.8% | 14.2% | 14.5% | 16.1% | 16.3% | 16.1% |
| Q3 | 14.9% | 15.1% | 14.8% | 15.2% | 15.2% | 14.9% | 14.6% | 14.7% | 15.1% |
| Q4 | 20.9% | 21.1% | 21.0% | 20.4% | 19.9% | 20.0% | 17.8% | 18.6% | 18.3% |
| Q5 (Highest) | 39.4% | 39.3% | 39.0% | 35.6% | 35.2% | 34.6% | 32.0% | 29.9% | 28.6% |

Values are mean ± SD, or %

IPAQ = International Physical Activity Questionnaire

**eTable 5. Baseline characteristics by glycemic status and polygenic risk group of ischemic stroke.**

|  | **Low PRS** | | | **Intermediate PRS** | | | **High PRS** | | |
| --- | --- | --- | --- | --- | --- | --- | --- | --- | --- |
|  | **HbA1c < 5.6%** | **HbA1c 5.7-6.4%** | **HbA1c ≥ 6.5%** | **HbA1c < 5.6%** | **HbA1c 5.7-6.4%** | **HbA1c ≥ 6.5%** | **HbA1c < 5.6%** | **HbA1c 5.7-6.4%** | **HbA1c ≥ 6.5%** |
| Total, No. | 89338 | 14586 | 4225 | 87073 | 15165 | 5233 | 84480 | 15357 | 6386 |
| Age, mean (SD), y | 56.2 ± 8.2 | 56.1 ± 8.2 | 55.9 ± 8.2 | 60.1 ± 7.1 | 60.1 ± 7.0 | 60.0 ± 7.1 | 59.9 ± 7.1 | 59.6 ± 7.2 | 59.6 ± 7.3 |
| Female | 47.2% | 47.3% | 47.0% | 49.5% | 49.8% | 49.3% | 65.1% | 64.6% | 64.7% |
| Ethnicity |  |  |  |  |  |  |  |  |  |
| White | 95.7% | 96.5% | 96.3% | 89.4% | 91.6% | 91.6% | 84.3% | 88.2% | 88.2% |
| Black | 1.1% | 0.7% | 0.8% | 3.6% | 2.5% | 2.3% | 4.1% | 3.0% | 2.3% |
| Asian | 1.6% | 1.3% | 1.5% | 4.4% | 3.8% | 3.8% | 8.0% | 6.7% | 6.6% |
| Mixed/other | 1.6% | 1.4% | 1.4% | 2.6% | 2.2% | 2.3% | 3.6% | 2.1% | 2.9% |
| BMI, mean (SD) | 26.5 ± 4.2 | 26.8 ± 4.3 | 27.2 ± 4.5 | 28.6 ± 5.1 | 28.9 ± 5.2 | 29.4 ± 5.4 | 31.0 ± 5.7 | 31.7 ± 5.9 | 31.9 ± 5.9 |
| Alcohol |  |  |  |  |  |  |  |  |  |
| Non | 6.1% | 6.4% | 6.7% | 10.4% | 10.5% | 10.8% | 15.7% | 15.2% | 15.4% |
| 1-2 times/week | 45.6% | 46.4% | 47.0% | 50.4% | 51.2% | 51.6% | 52.5% | 53.4% | 55.6% |
| ≥3 times/week | 48.2% | 47.2% | 46.3% | 39.2% | 38.3% | 37.6% | 31.8% | 31.3% | 29.0% |
| Smoking |  |  |  |  |  |  |  |  |  |
| Non- | 57.2% | 56.3% | 55.3% | 50.2% | 47.9% | 46.0% | 48.3% | 44.6% | 44.2% |
| Ex- | 34.0% | 34.5% | 34.6% | 36.2% | 37.4% | 38.1% | 40.3% | 42.8% | 43.4% |
| Current- | 8.8% | 9.2% | 10.1% | 13.7% | 14.7% | 15.8% | 11.5% | 12.6% | 12.4% |
| Hypertension | 16.2% | 22.5% | 30.3% | 30.3% | 39.4% | 48.7% | 52.8% | 61.1% | 70.4% |
| Dyslipidemia | 9.0% | 10.6% | 12.6% | 21.5% | 25.7% | 28.4% | 39.8% | 42.6% | 45.3% |
| Heart failure | 1.9% | 2.0% | 2.1% | 2.8% | 3.3% | 3.5% | 4.7% | 4.1% | 5.2% |
| Peripheral artery disease | 0.3% | 0.3% | 0.4% | 0.5% | 0.7% | 0.9% | 0.9% | 1.0% | 1.2% |
| CKD or ESRD | 0.7% | 0.8% | 0.9% | 1.5% | 1.8% | 2.2% | 3.1% | 3.5% | 4.1% |
| IPAQ |  |  |  |  |  |  |  |  |  |
| Low | 17.8% | 18.0% | 18.0% | 20.4% | 20.9% | 21.4% | 28.7% | 29.6% | 29.5% |
| Intermediate | 41.3% | 40.6% | 40.4% | 41.4% | 41.1% | 40.5% | 40.5% | 38.7% | 39.9% |
| High | 40.9% | 41.4% | 41.5% | 38.2% | 38.0% | 38.1% | 30.8% | 31.7% | 30.7% |
| Economic status |  |  |  |  |  |  |  |  |  |
| Q1 (Lowest) | 11.6% | 11.9% | 12.8% | 14.5% | 15.6% | 16.9% | 19.4% | 21.8% | 21.8% |
| Q2 | 12.7% | 12.9% | 12.7% | 13.9% | 14.3% | 14.5% | 16.7% | 15.0% | 16.7% |
| Q3 | 14.9% | 14.9% | 14.9% | 15.5% | 15.0% | 14.9% | 14.4% | 14.8% | 15.0% |
| Q4 | 21.0% | 21.2% | 20.8% | 20.6% | 19.9% | 19.4% | 19.0% | 18.0% | 18.0% |
| Q5 (Highest) | 39.7% | 39.1% | 38.7% | 35.5% | 35.2% | 34.2% | 30.5% | 30.3% | 28.5% |

Values are mean ± SD, or No. (%)

IPAQ = International Physical Activity Questionnaire

**eTable 6.** Cumulative incidence and incidence rates for outcomes according to polygenic risk score group.

|  | **Atrial fibrillation** | | | | **Coronary artery disease** | | | | **Ischemic stroke** | | | |
| --- | --- | --- | --- | --- | --- | --- | --- | --- | --- | --- | --- | --- |
| **Polygenic risk score*** | **No. at risk** | **No. of events /Person-year** | **IR per 1,000 person-years** | **HR**  **(95% CI)** | **No. at risk** | **No. at risk** | **IR per 1,000 person-years** | **HR**  **(95% CI)** | **No. at risk** | **No. of events /Person-year** | **IR per 1,000 person-years** | **HR**  **(95% CI)** |
| Low | 105509 | 3607/1213806 | 2.97 | Reference | 106921 | 1835/1239814 | 1.48 | Reference | 108149 | 876/1256333 | 0.70 | Reference |
| Intermediate | 104702 | 5404/1196060 | 4.52 | 1.52  (1.46-1.59) | 105642 | 3390/1218236 | 2.78 | 1.86  (1.75-1.97) | 107471 | 1113/1248041 | 0.89 | 1.21  (1.11-1.32) |
| High | 103120 | 8386/1161716 | 7.22 | 2.46  (2.37-2.56) | 103135 | 5712/1175218 | 4.86 | 3.26  (3.09-3.44) | 106223 | 1500/1229248 | 1.22 | 1.57  (1.45-1.71) |

*Polygenic risk score was categorized as low (tertile 1), intermediate (tertile 2), or high (tertile 3).

Adjusted for age, sex, ethnicity, body mass index, smoking, alcohol, diabetes, hypertension, dyslipidemia, heart failure, peripheral artery disease, chronic kidney disease, end stage renal disease, IPAQ-PA, and economic status.

HR = Hazard ratio, IR = Incidence rate.

**eFigure 1.** Incidence rate of atrial fibrillation, coronary artery disease and ischemic stroke according to the percentile of the polygenic score.

**
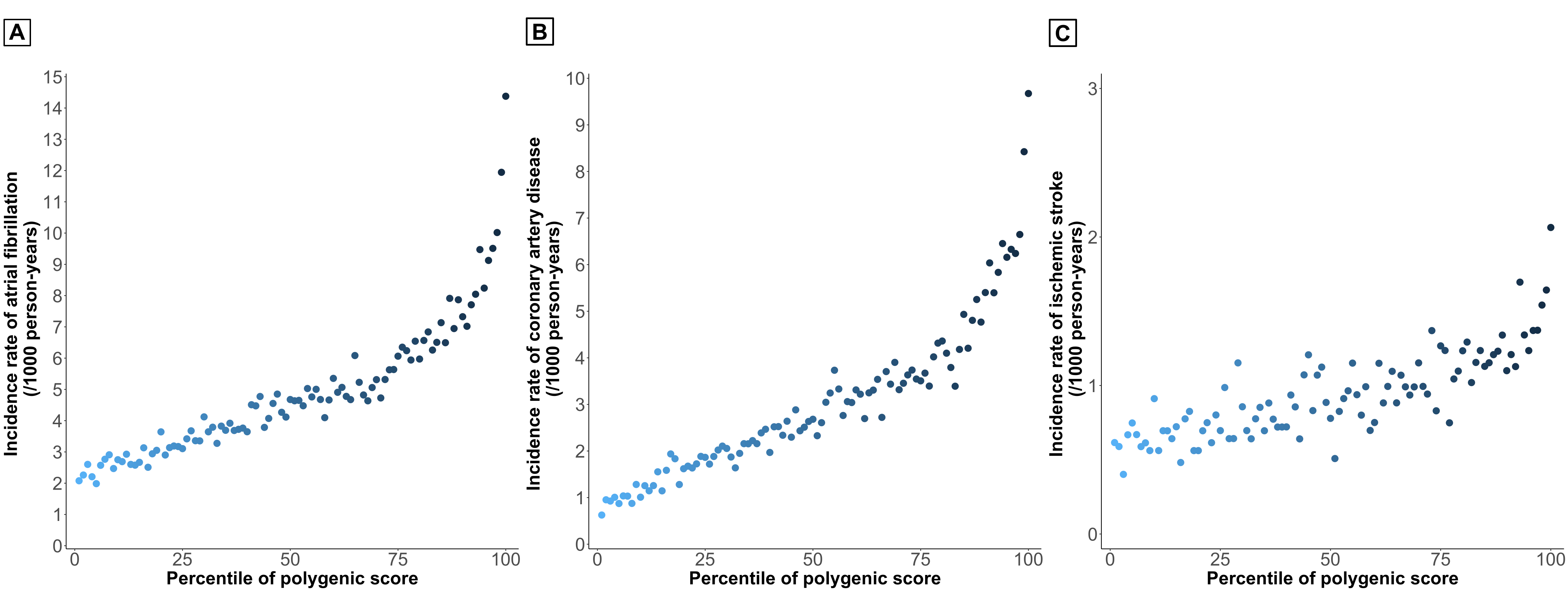
**

**eFigure 2.** Hazard ratios for atrial fibrillation, coronary artery disease and ischemic stroke by polygenic risk score and clinical risk factors.

**
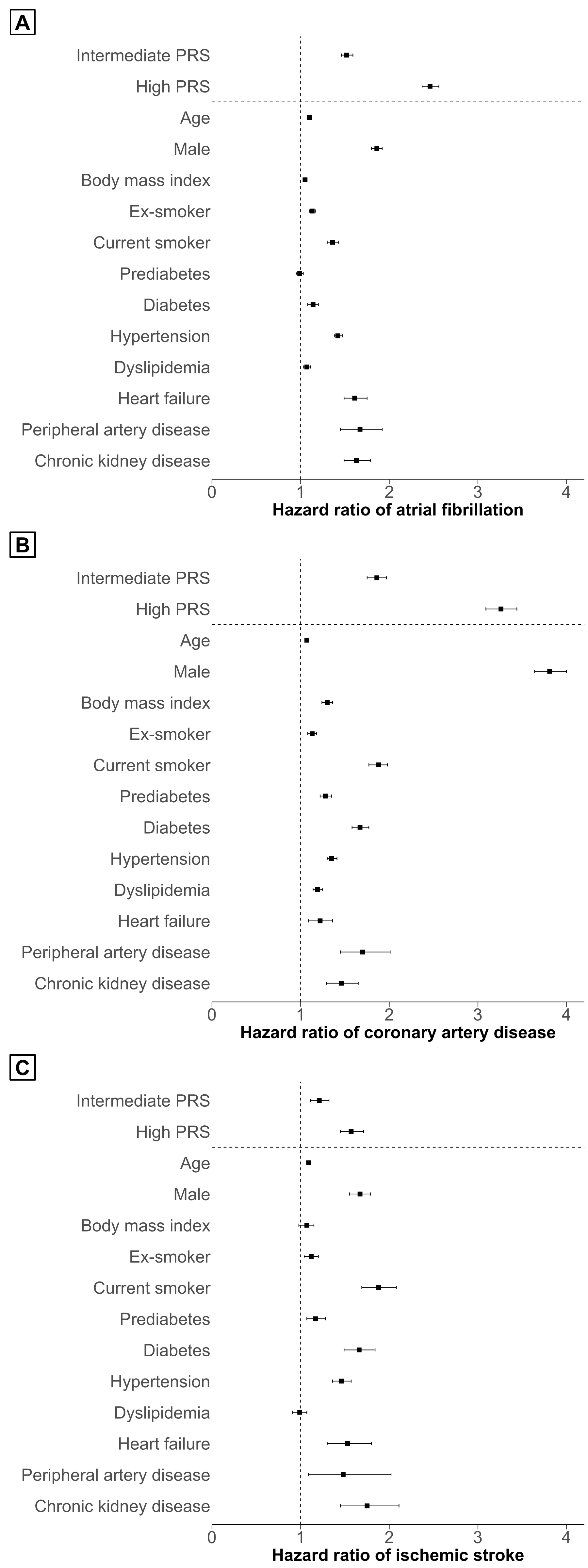
**

Adjusted for age, sex, ethnicity, body mass index, smoking, alcohol, diabetes, hypertension, dyslipidemia, heart failure, peripheral artery disease, chronic kidney disease, end stage renal disease, IPAQ-PA, and economic status.

**eFigure 3.** Associations of hemoglobin A1c with atrial fibrillation, coronary artery disease and ischemic stroke by polygenic risk score, categorized as low (quintile 1), intermediate (quintile 2-4), or high (quintile 5): sensitivity analysis.


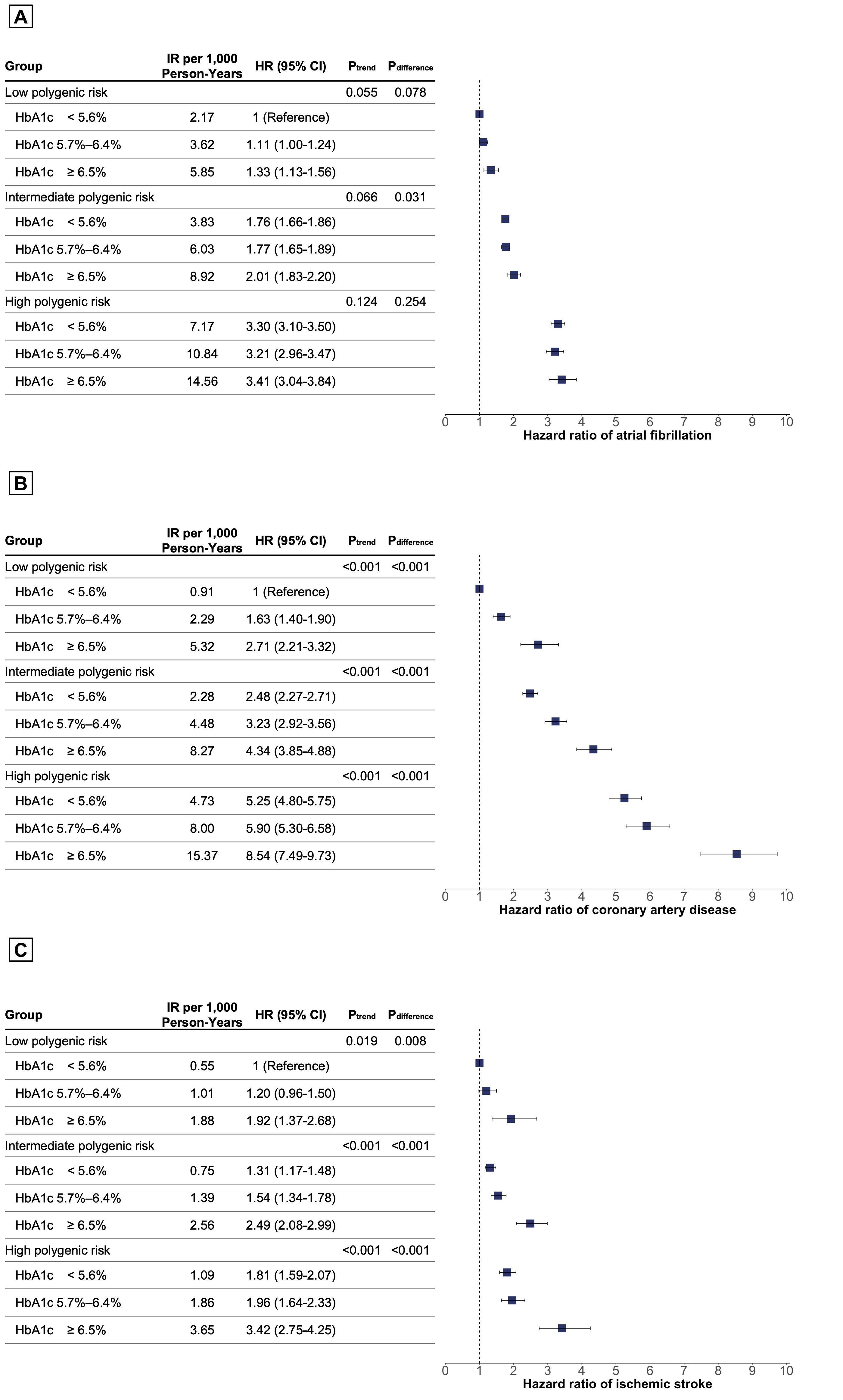


Adjusted for age, sex, ethnicity, body mass index, smoking, alcohol, hypertension, dyslipidemia, heart failure, peripheral artery disease, chronic kidney disease, end stage renal disease, IPAQ-PA, and economic status.

**eFigure 4.** Associations of hemoglobin A1c with atrial fibrillation, coronary artery disease and ischemic stroke by polygenic risk score in the European population.


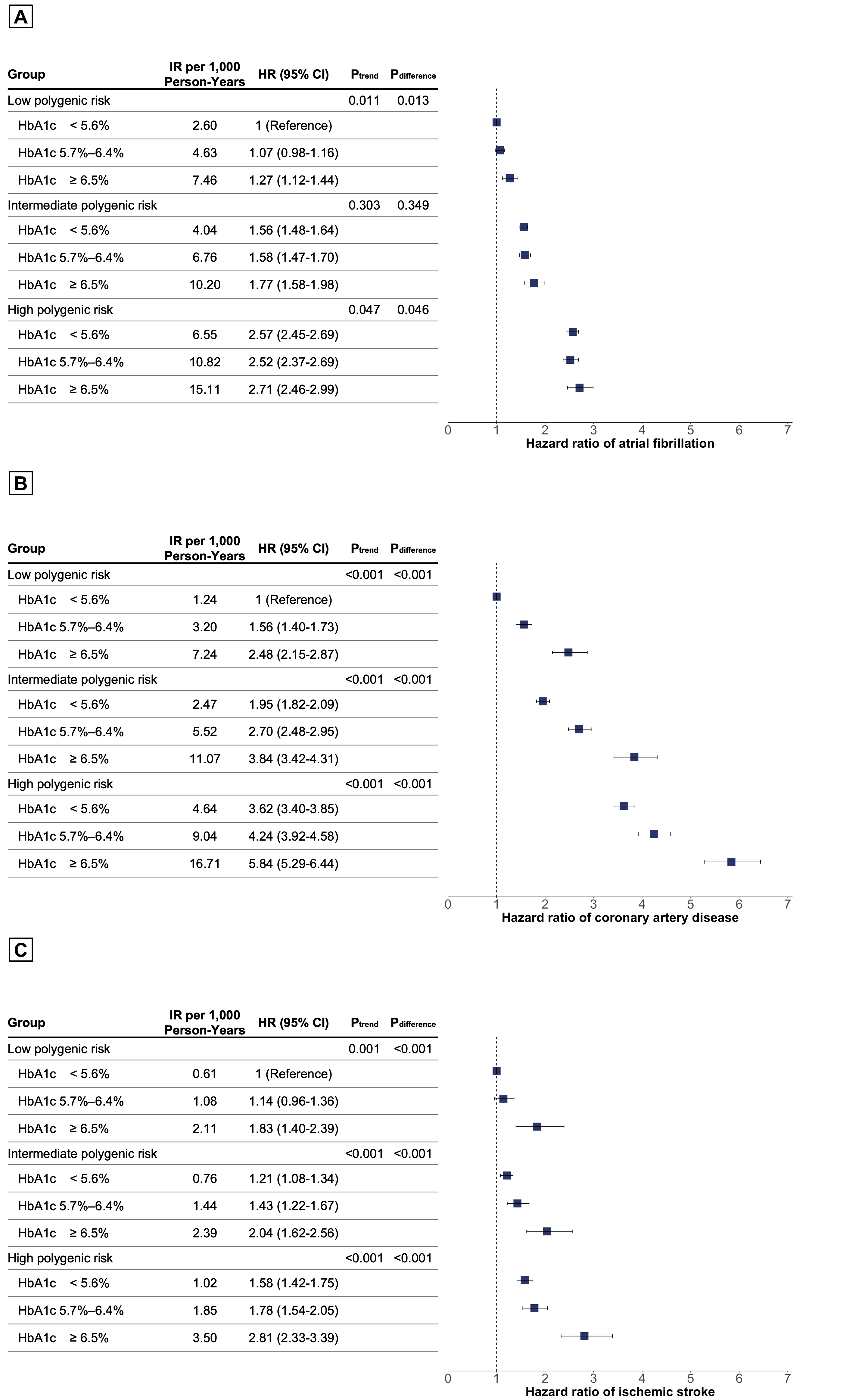


Adjusted for age, sex, body mass index, smoking, alcohol, hypertension, dyslipidemia, heart failure, peripheral artery disease, chronic kidney disease, end stage renal disease, IPAQ-PA, and economic status.

Hazard ratios (HRs) are provided with 95% CIs. The vertical line indicates the reference value of 1.

**eFigure 5.** Nonlinear dose–response analysis of hemoglobin A1c and the risk of atrial fibrillation, coronary artery disease and ischemic stroke, by polygenetic risk score tertiles in the European and Asian population.


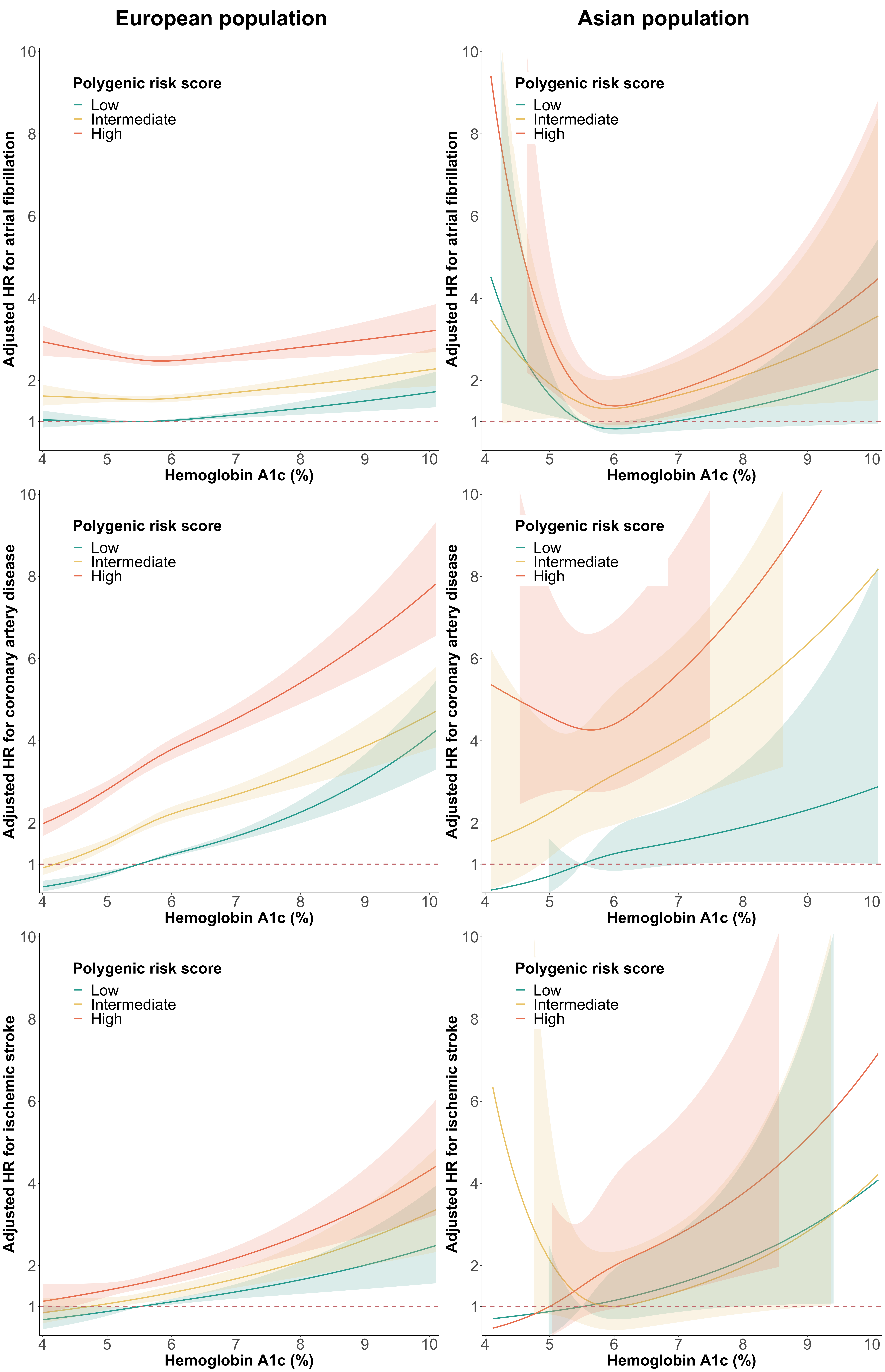


The model is centered at 5.5% HbA1c with knots at 5.0%, 5.7%, and 6.5% HbA1c.

**eFigure 6.** Risk of incident atrial fibrillation, coronary artery disease and ischemic stroke associated with polygenic risk score and hemoglobin A1c category, stratified by sex.


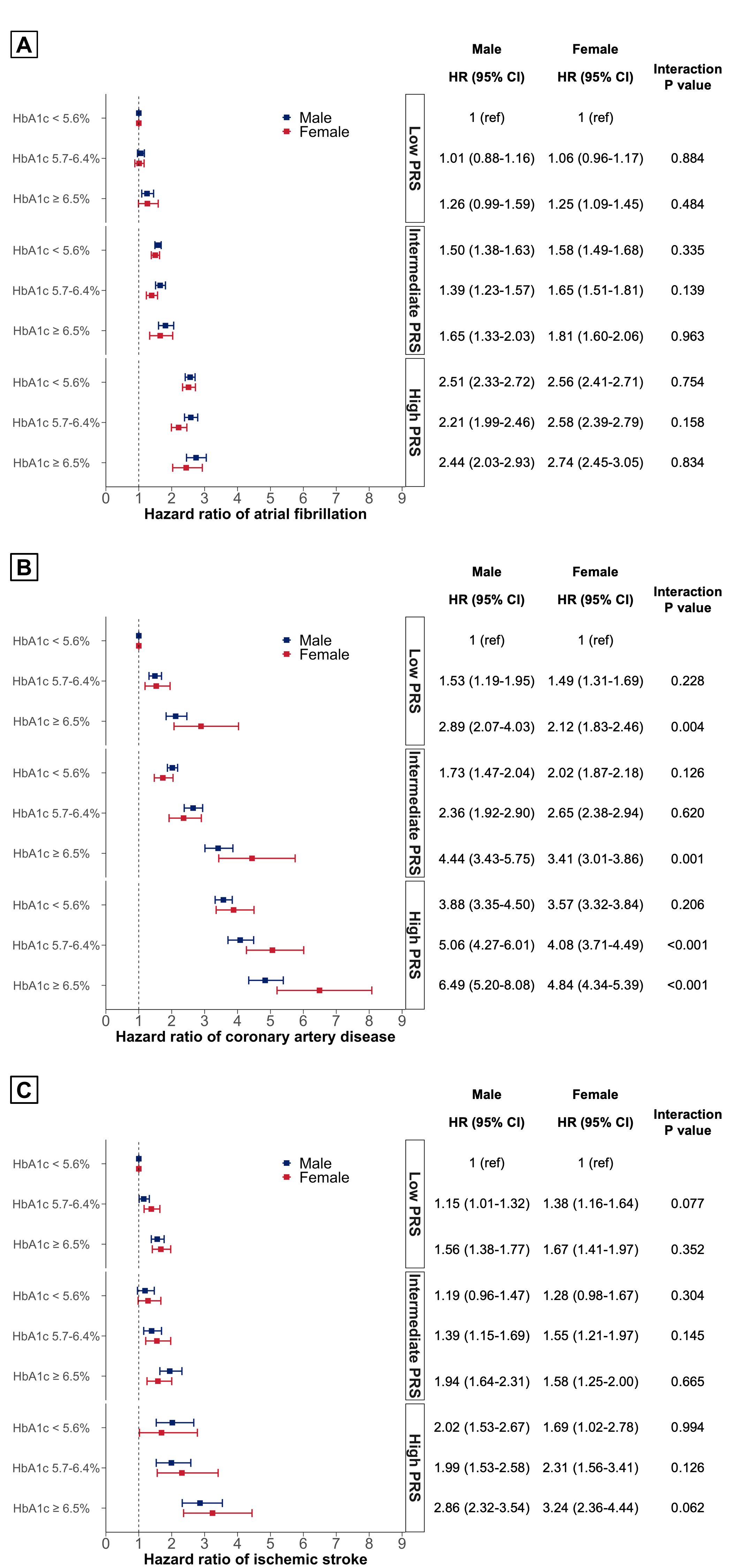

Supplement: Supplementary file 1 — Supplementary Material 1 [file 12933_2023_2021_MOESM1_ESM.docx]
